# A monocentric prospective study investigating digital engagement among geriatric hospital patients

**DOI:** 10.1186/s12877-025-05953-2

**Published:** 2025-05-20

**Authors:** Julia Göbel, Anna Kordowski, Jennifer Kasper, Martin Willkomm, Christian Sina

**Affiliations:** 1 Institute of Nutritional Medicine, University Hospital of Schleswig-Holstein, Campus Lübeck and University of Lübeck, Lübeck, Germany; 2 Research Group Geriatric Lübeck, Hospital “Rotes Kreuz Lübeck - Geriatriezentrum”, Lübeck, Germany; 3 Fraunhofer Research Institution of Individualised and Cell-Based Medical Engineering (IMTE), Lübeck, Germany

**Keywords:** Aging society, Geriatric health care, Digital participation, Wearables, Usability, Feasibility

## Abstract

**Background:**

The aging of society drives a rising demand for geriatric healthcare due to increased care needs and extended hospital stays in old age. Despite strained social security systems, ensuring high-quality medical care requires innovative solutions. Digitalization could be one of them, however older people, who are less digitally active, may not fully recognize its benefits. This study aims to assess digital participation among geriatric hospital patients and their views on continuous vital sign monitoring using wearables.

**Methods:**

The survey was conducted at the geriatric hospital “Krankenhaus Rotes Kreuz Lübeck – Geriatriezentrum” to assess the digital participation of higher frailty patients requiring increased care. The questioning occurred between February 13th and March 10th, 2023. The questionnaire included demographic questions, questions about digital participation and digital skills, opinions on continuous monitoring, and a reflection on the impact of the coronavirus pandemic on internet use.

**Results:**

Of the 201 consecutively admitted patients, 52 were excluded from participation in the study based on the inclusion/exclusion criteria, mostly due to illness. Of the remaining 149 invited patients, 66 (44.2%) agreed to be interviewed, mostly females (76%) with an average age of 81.2 years (SD = 7.1). As a result, 68.2% of participants reported online activity, whereby females and those with low education or high age (*p* = 0.027) were offline more often. On average, 1–2 internet-enabled devices were used. Continuous vital sign monitoring was favoured by 32 participants and 61 expressed no concerns.

**Conclusion:**

Our findings align with previous studies involving participants of comparable age, indicating comparable results, apart from disease-related participation restrictions. However, the significant proportion of patients who did not want to participate (55.7%) and the analysis of the reasons for nonparticipation suggest that the actual number of geriatric patients who do not engage online is higher. While this does not necessarily imply a complete rejection of digital products by this demographic, it highlights the need for greater emphasis on usability, feasibility, and clarification in future endeavors.

**Supplementary Information:**

The online version contains supplementary material available at 10.1186/s12877-025-05953-2.

## Background

Demographic change is leading to an increasingly aging society, resulting in a significant strain on geriatric patient care. The burden of rapid population aging is evident in the rising incidence of comorbidities, dementia, and chronic diseases in older adults [1], which contribute to poorer health outcomes and increased healthcare costs. These health issues are prevalent among geriatric patients, making them more vulnerable to loss of independence and requiring extensive care [2]. At the same time, a shortage of skilled workers in this field exacerbates the gap in care provision [3]. To address these challenges and maintain the quality of medical and nursing care, more effective solutions must be developed [4].

Concomitantly, technical progress is increasing rapidly, which is why digitalization harbors substantial potential to relieve the burden on the health system. This potential includes advancements such as wearables [5], legal regulatory frameworks exemplified by the Digital Health Care Law [6], and decision-making processes propelled by artificial intelligence [7]. The advent of the coronavirus pandemic in 2020 has additionally accelerated the momentum of digital transformation [8]. Despite the potential benefits, older generations have yet to fully experience the advantages of digitization. However, these groups could greatly benefit from digital offerings. For instance, online shopping can help compensate for reduced mobility in older individuals [9], while digital print media can provide a solution for those with visual impairments [10].

Acquiring digital competence is increasingly important for social participation [11]. Since much information is now available with internet access and more services can only be accessed via the internet, the use of the internet is necessary to communicate [12].

There are also various digital offers in fitness tracking and lifestyle, where the focus has been more on prevention, knowledge transfer and general health promotion. An illustrative example of technological advancements is the rise of wearables, compact and portable sensors commonly found in wristbands or smartwatches. These devices are often paired with health-oriented applications and are predominantly embraced by health-conscious individuals. It is already possible to collect medically relevant data. For example, continuous glucose monitoring can be facilitated by the help of a tissue patch [13] and cardiac arrhythmias can be detected via a smartwatch [14, 15]. Through the continuous monitoring of various vital signs, a complete health chronicle can be documented. The healthcare benefits greatly from this dynamic, as constant monitoring of critical signs offers the potential for early detection and intervention of diseases. This, in turn, has the potential to shorten or even reduce hospital stays [12]. These more efficient treatment options could also save medical costs [16].

To date, wearables have been used more by younger generations, as a smartphone with the internet is usually a prerequisite for wearables [16]. It is estimated that only one-third of the German population over 80 uses a smartphone [17]. This means that many older people still need to fulfil the necessary conditions for using a wearable. To understand the functions of technical innovations, digital competence is a prerequisite. However, only 14% of people over 76 have basic digital skills [18]. In addition, just one in two of the over 60s and only one in five of the over 80s personally see a benefit for themselves in digitisation [8]. This shows that the older generations have much to do regarding digital participation and that the use of digital applications is rather foreign to everyday life.

For precisely this context this study was designed. The purpose of this study was twofold: firstly, to gain insights into the digital participation of geriatric hospital patients, and secondly, to assess their willingness to integrate automated, digital devices into their daily clinical routines. By conducting a comprehensive inquiry, this study aimed to determine the extent of digital participation among geriatric participants and their overall openness to digital solutions.

## Methods

### Study design

The study was designed as monocentric prospective human study investigating the digital engagement of patients admitted to a geriatric hospital via a questionnaire. The study center was the geriatric hospital in Lübeck, Germany (Krankenhaus Rotes Kreuz Lübeck – Geriatriezentrum). The ethics committee of the University of Lübeck approved this study on January 24th, 2023 (file number: 2023 − 152). The study was conducted as part of a master thesis between February 13th and March 10th, 2023.

### Study population

The study population comprised of patients of the geriatric hospital. Participants were identified and enlisted during their hospitalization, whether on a stationary or a partial inpatient stay. All participants gave informed consent to their anonymized data being used for scientific purposes.

The patient must have been able to give written consent to participate in the survey. In addition, knowledge of German and sufficient hearing ability to understand the questions well, were necessary to participate in this study. The absence of substantial cognitive impairment was paramount, established by a requisite SIS score (Six-Item-Screen Test) of four or higher [19]. Non-fulfilment of all inclusion criteria led to exclusion.

#### Sample calculation

The survey, conducted as part of a preliminary study, did not involve precise sample size planning but estimated participation based on patient numbers. With the geriatrics center hosting approximately 150 inpatients and 50 outpatients, it was expected that about 100 participants could be surveyed over a four-week period. This estimate assumed five patients per day, five days a week.

Given that more patients would likely be screened than eligible for participation, the four-week period was selected to account for patient turnover, as geriatric hospital patients are typically admitted for three weeks.

### Implementation

First, patient records were employed to determine eligibility for obtaining informed consent, primarily by verifying the absence of a more severe cognitive disorder. The SIS score, performed as a routine assessment during patient admission, served as the determinant criterion in this regard. Consequently, no additional cognition tests were administered within this study’s framework.

Next, all patients with an SIS score exceeding three were visited on the hospital wards and the inclusion and exclusion criteria were verified. Upon inclusion, the survey’s topic was explained, and patients were provided with both verbal and written explanations regarding the procedure and the management of collected data. Afterwards, the patient could sign the consent form and participate in the survey.

### Interview questions

The questionnaire provided to the patients was developed within the framework of this study. It consisted of own and modified questions from already existing questionnaires on the topic of digitalisation among senior citizens [20] and digital participation among very old people [17], as well as from the D21 Digital-Index [8]. The complete questionnaire is attached as Additional file 1.

The first part of the survey collected demographic data such as gender, year of birth, educational qualification, and profession. The subsequent part encompassed the digital participation of the participants concerning their use of the internet and digital devices, their digital skills, and their motivation to be digitally active. In the third part, the example of continuous monitoring (cM) was used to obtain the participant’s opinions on how this type of monitoring would be accepted. For this purpose, we designed our questions. The final set of questions offered the participant a short reflection on the topic and showed to which extent the coronavirus pandemic influenced the participant’s internet use. In addition to the survey questions, the participants were asked in the form of free text for additional information (e.g., if they expressed concerns about cM, they were asked why). If this information was provided, it was also analyzed.

### Statistical analysis

The obtained results underwent a descriptive analysis facilitated by Microsoft Excel. This analytical approach involved the calculation of either absolute counts or the corresponding percentages within distinct participant groups. Further, mean value calculations, coupled with corresponding standard deviations, were executed. The exploration of potential statistical associations between two categorical variables was conducted utilizing the Chi-Square-Test test, facilitated by the Statistical Package for the Social Sciences (SPSS).

## Results

### Study population

The survey was conducted over four weeks, from February 13 to March 10, 2023. During this period, 201 patients were screened, of whom 52 did not meet the inclusion criteria. Among the remaining 149 potential participants, 66 consented to be interviewed, representing approximately 44% of eligible patients.

**Fig. 1:**
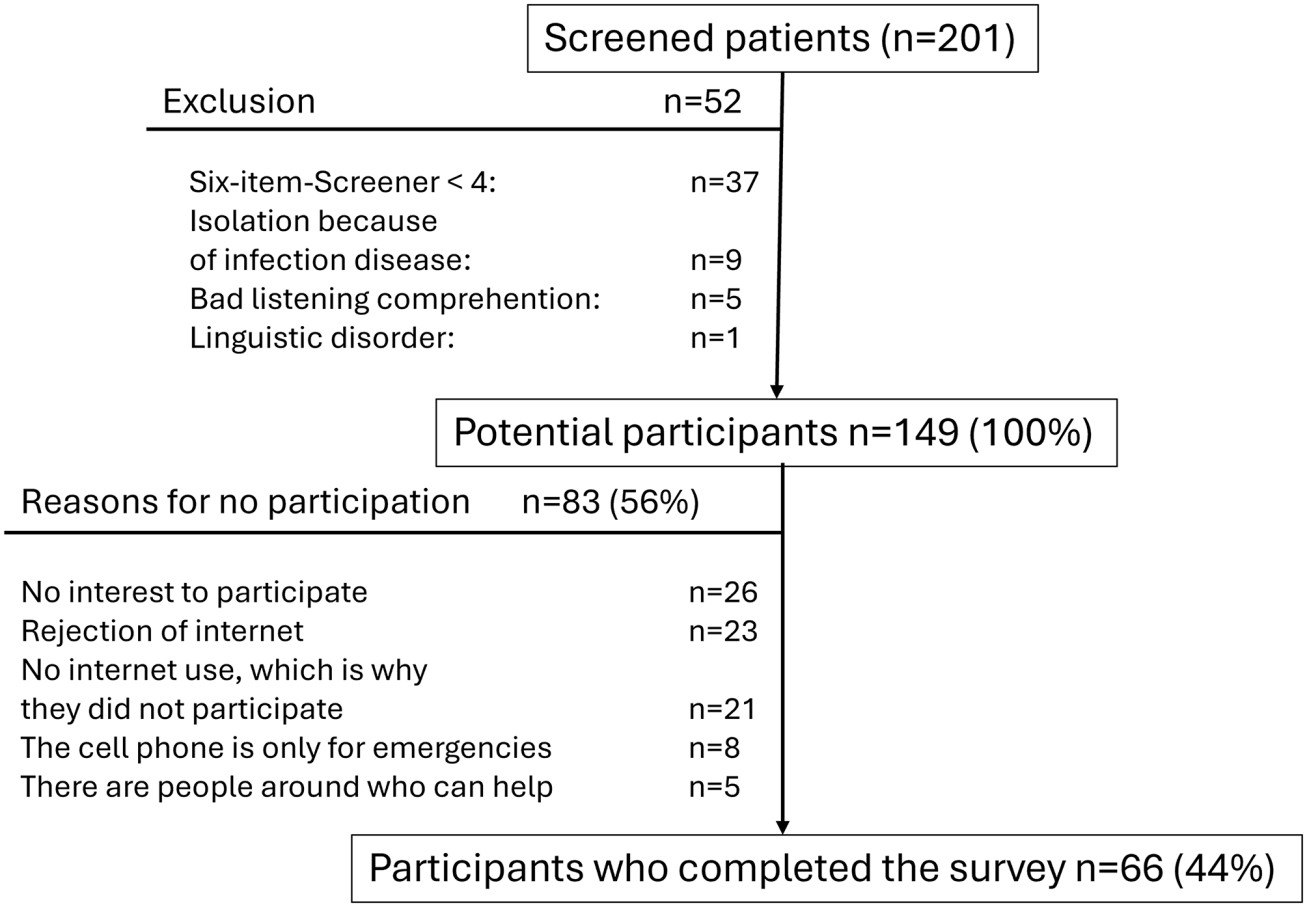
Flow Diagram of Patient Population

Of the 83 nonparticipants, 23 patients expressed a general disagreement with the internet, 21 declined because they did not use the internet, and 8 stated that their cell phone was only used for emergencies. Additionally, 26 patients had no interest in participating in the survey, and 5 indicated they had people supporting them. The reasons for nonparticipation were collected in free-text form and subsequently categorized into these five groups (Fig. 1).

Among the 66 participants, 76% were female, with a mean age of 81.2 ± 7.1 years, ranging from 64 to 98 years. Regarding age distribution, 56% of participants were between 80 and 89 years old. Together with those aged 90–99 years, two-thirds of the participants were classified as very old. Importantly, the comparison of age and gender between participants and nonparticipants showed a similar distribution, suggesting that the participant group may be representative of geriatric hospital patients in terms of these characteristics (Table 1).

**Table 1:** Demographics of participants

	Participants (*n* = 66)	Nonparticipants (*n* = 83)	
**gender**:	n (percentage)	n (percentage)	
female	50 (76%)	55 (66%)	Chi^2^*p* = 0,207
male	16 (24%)	28 (34%)	
**age**: average ± SD	81.2 ± 7.1	80,5 ± 7.0	T-Test *p* = 0,665
**Age distribution**:	n (percentage)	n (percentage)	
60–69	5 (8%)	6 (7%)	
70–79	18 (27%)	26 (31%)	
80–89	37 (56%)	46 (56%)	
90–99	5 (8%)	4 (6%)	
**Highest education**:	n (percentage)	n (percentage)	
None/ low education	32 (48%)		
Medium education	26 (39%)		
High education	8 (12%)		

Please note that, as a result of rounding the results, the total may not always add up to 100%

### Digital participation of geriatric hospital patients

Among the 66 patients who participated in the survey, 45 individuals reported active internet usage, while 21 participants were classified as non-users, constituting the “offliner” category. This indicates that 68.2% of the participants were active online.

However, a closer examination of the reasons for nonparticipation in the survey of nonparticipants revealed that at least 52 of these patients did not use the internet and could also be categorized as offliners. For the remaining 31 nonparticipants, it was not possible to determine whether they were onliners or offliners.

In conclusion, out of the 149 potential participants, 30% were onliners, 49% were offliners, and 21% could not be categorized into either group.

**Fig. 2:**
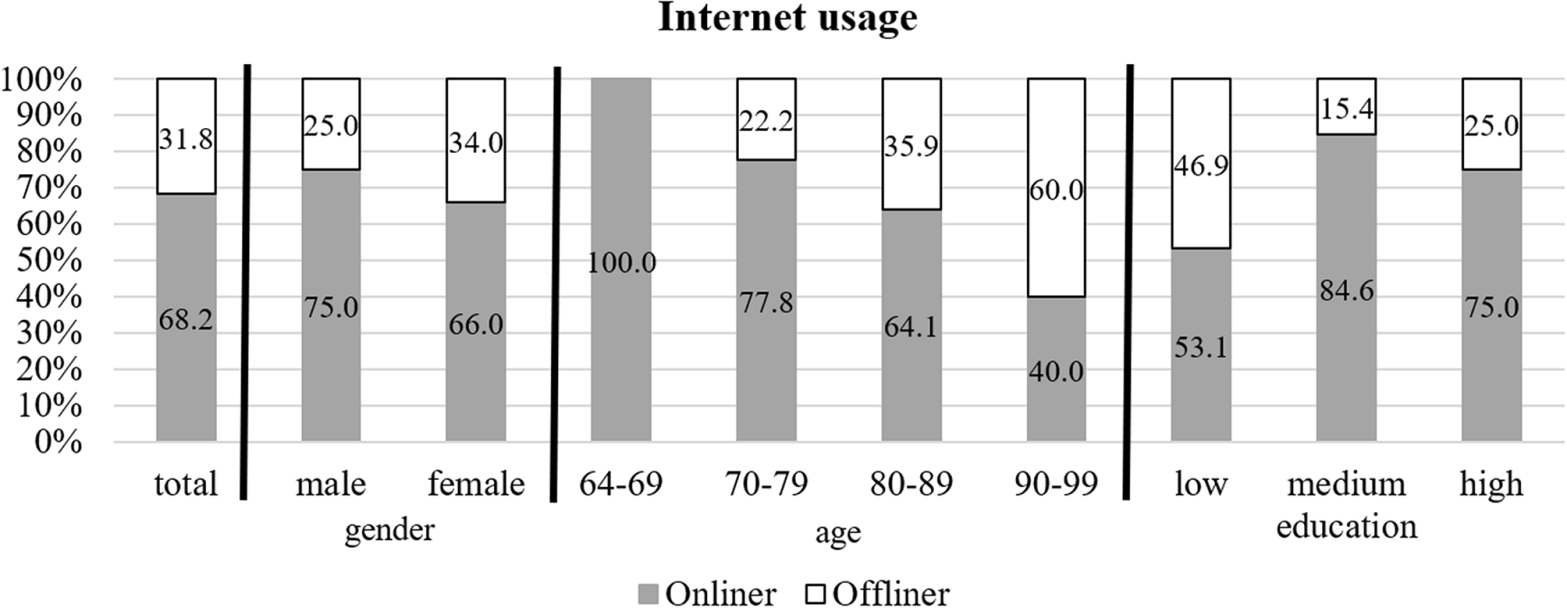
Relationship among internet use and gender, age, or level of education in *n* = 66 participants

Notably, internet usage among participants was influenced by gender, age, and level of education. Male participants and those with higher levels of education were more frequently among the onliners compared to their female and older counterparts (Fig. 2). Additionally, a statistically significant association was found between internet usage and age (χ² = 4.89, *p* = 0.027).

#### Numerous factors contributing to offline status

**Fig. 3:**
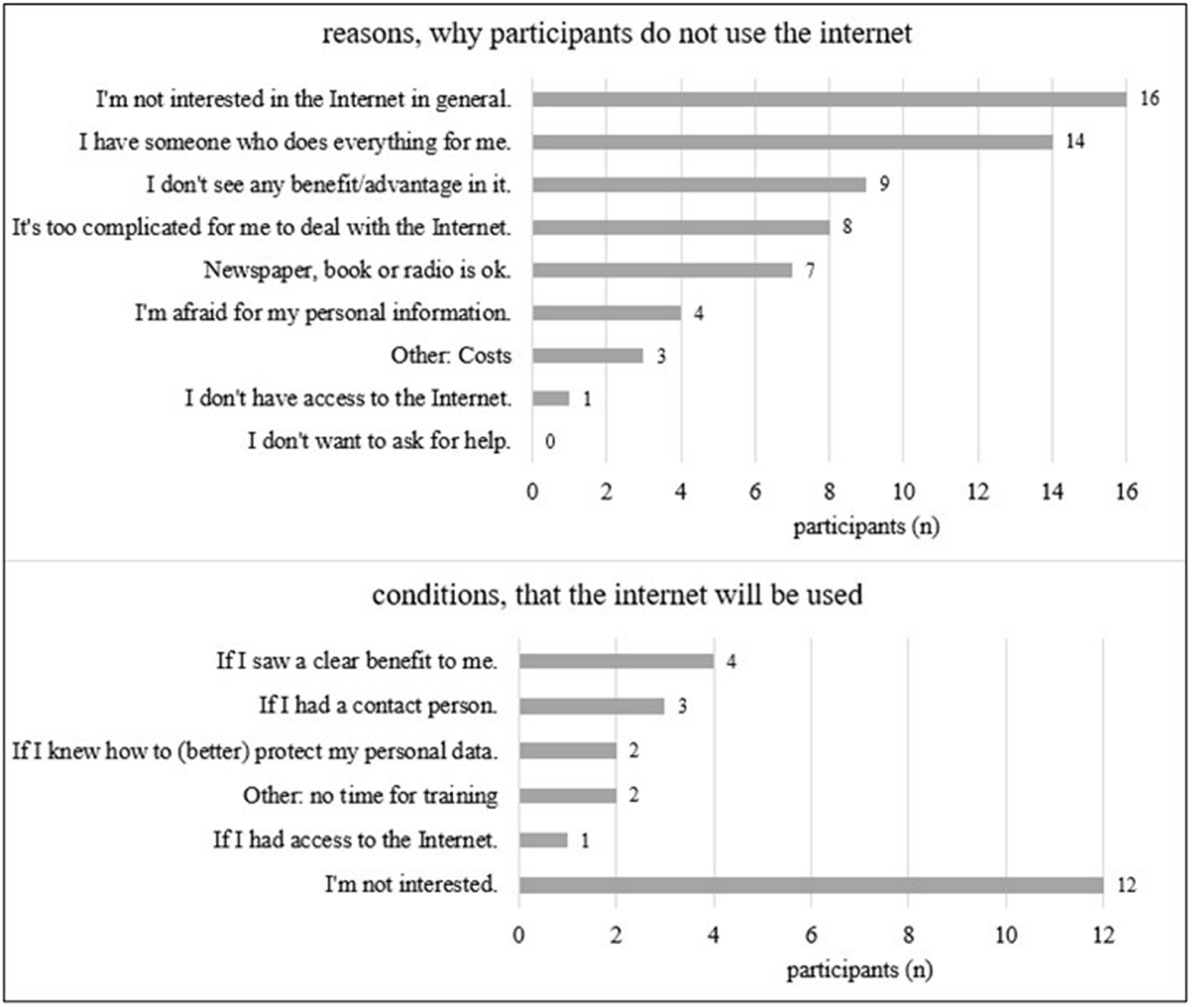
Reasons for offline status among 21 out of 66 participants and factors that could facilitate their transition to online engagement

To investigate the underlying motivations for offline status among 21 out of 66 participants, an in-depth analysis was conducted among those who did not use the internet. These participants provided reasons for abstaining from online activities and shared insights on the conditions under which they might adopt internet usage (Fig. 3). Notably, more reasons were cited for avoiding internet use than for potential adoption.

It is worth mentioning that nonparticipants provided reasons for not participating in the survey, which partially overlap with the reasons given by participants for not engaging with the internet. However, since nonparticipants could not be interviewed in detail, their responses were not included in this analysis. Therefore, the findings are based solely on the answers of the 21 participants who completed the survey.

#### An average of 1–2 internet-enabled devices were used

**Fig. 4:**
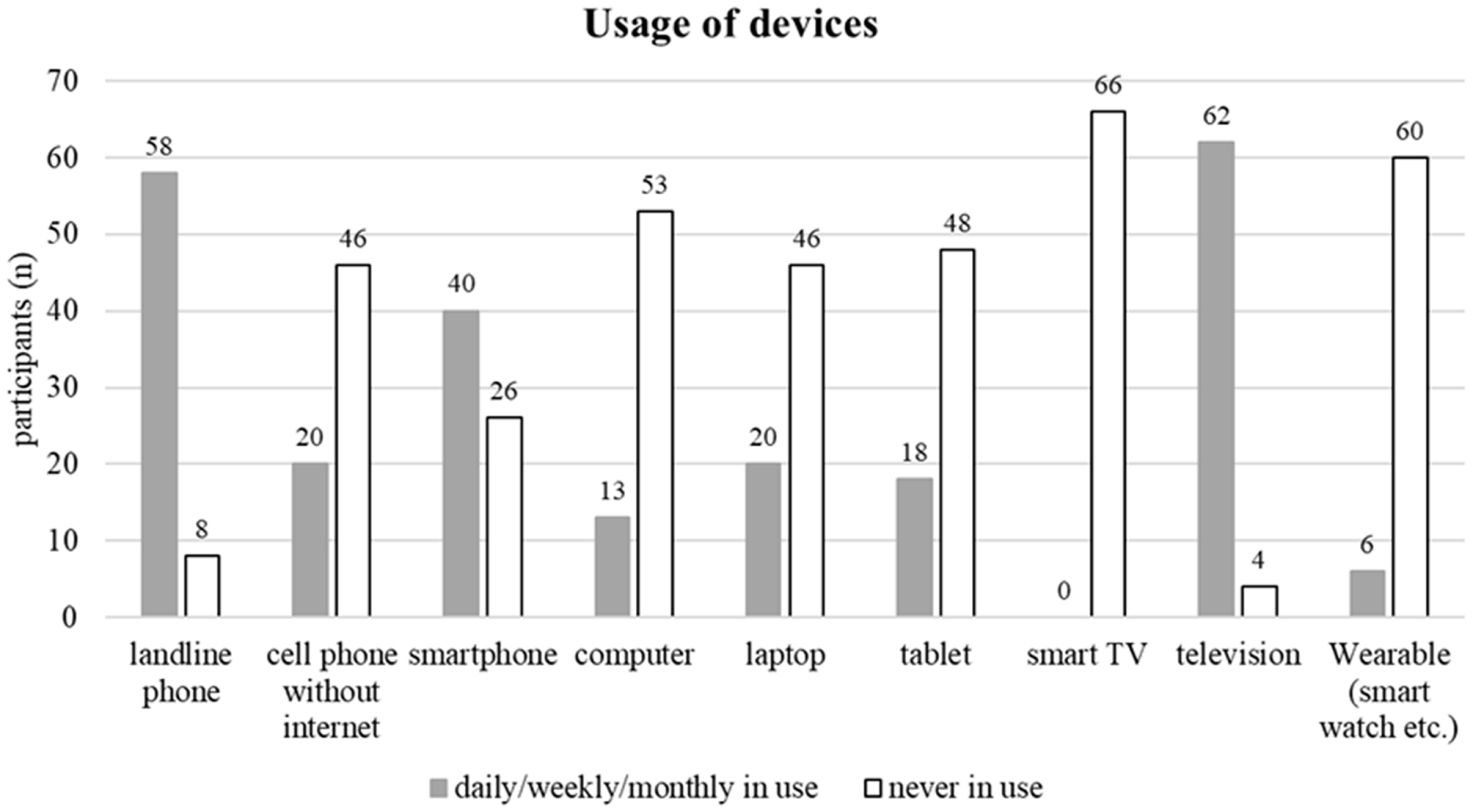
Overview of device usage

The survey’s findings of all 66 participants indicated an average usage of 1–2 internet-enabled devices per participant. Figure 4 provides insights into the range of devices privately employed by the participants. Each stated using at least one of the following technical devices: landline phone, cell phone without internet, smartphone, computer, laptop, tablet, TV, or wearable devices such as smartwatches. Notably, the highest number of privately employed devices by an individual was seven, while the majority reported using between three and four devices (4 × 1 gadget (G); 8 × 2G; 19 × 3G; 22 × 4G; 9 × 5G; 2 × 6G; 2 × 7G). If only the internet-enabled devices are considered, then most of the participants used 0–2 devices (18 × 0 gadgets (G); 16 × 1G; 20 × 2G; 9 × 3G; 1 × 4G; 2 × 5G; 0 × 6G; 0 × 7G). Moreover, six participants explicitly mentioned the usage of wearable devices. When queried further, three specified the employment of fitness watches, two acknowledged the use of step counter applications, and one participant utilized a tissue patch for blood glucose measurement together with its corresponding app.

#### Pervasive proficiency in device use and internet navigation, whereby the purpose of use varied greatly

A substantial majority of participants stated that they were (very) good at using devices for private purposes. Among onliners, 43 out of 45 reported being (very) competent in device usage, compared to 17 out of 21 offliners. While the onliners show a higher proportion of competence (95.6% vs. 81.0%), it is noteworthy that the offliners—who are not engaged with the internet—also report a high level of device competence.

In addition, 37 out of 45 onliners stated that they were (very) comfortable using the internet. Among various online applications, most onliners (33/45) used the internet for communication via messenger apps. Other activities included video conferencing (14/45), online shopping (13/45), and gaming apps (12/45), with approximately one-third of participants engaging in each. In contrast, streaming services (7/45) and healthcare applications (6/45) were less frequently used.

A closer look at onliners’ attitudes toward technology revealed that three-quarters expressed interest in technological devices. Furthermore, 37 out of 45 (82%) believed that digital devices could improve daily life, and 27 out of 45 (60%) indicated a willingness to explore video consultations with healthcare professionals. Additionally, 42 out of 45 (93%) felt they would benefit from technological progress. These figures were higher compared to offliners, with 9 out of 21 (42%) expressing interest in technology, 4 out of 21 (19%) believing in its potential to improve daily life, and 11 out of 21 (52%) open to video consultations.

Over half of the onliners demonstrated competence in digital skills, such as taking and sending pictures with their smartphone (38/45, 84%), completing online forms (29/45, 64%), and managing passwords (29/45, 64%).

Regarding the impact of the coronavirus pandemic on internet use, 38 out of 45 (84%) online users reported little to no rise in their usage, while 5 out of 45 (11%) experienced a moderate and 2 out of 45 (4%) reported a significant increase.

### Perceptions regarding continuous monitoring of vital signs via wearables

**Fig. 5:**
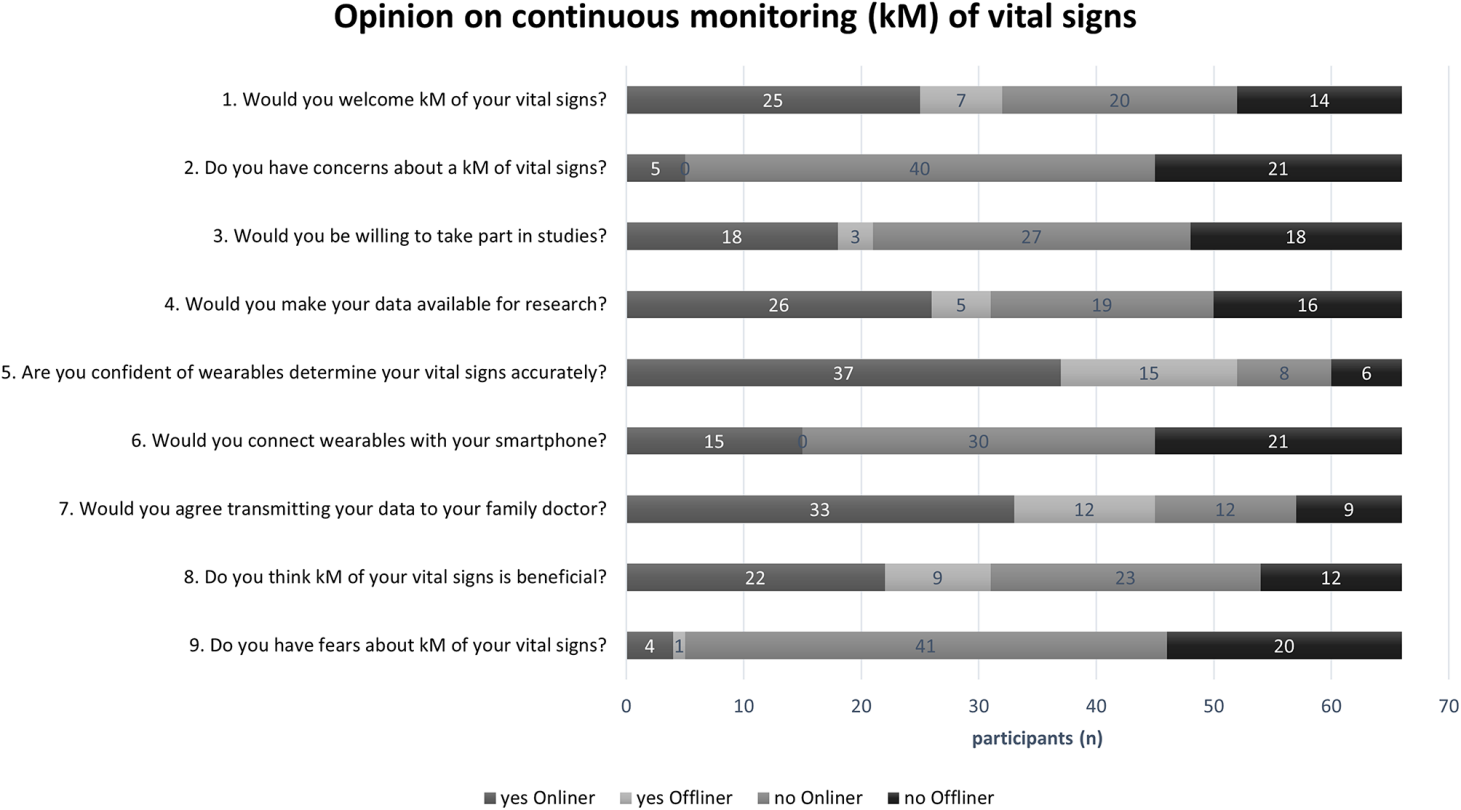
Opinion on continuous monitoring of vital signs

In the third part of the survey, participants were asked for their opinions on the topic of continuous monitoring (cM) of vital signs (Fig. 5). This section aimed to assess not only their initial stance but also the stability and reflectiveness of their opinions throughout the survey. To evaluate this, questions 1 and 8, as well as questions 2 and 9, addressed similar topics but were phrased differently. Both online and offline participants were interviewed, and the overall results, shown in Fig. 5, initially indicated relatively stable opinions across participants.

We specifically analyzed responses to questions 1 and 8, where 32 participants expressed a favorable stance toward continuous monitoring in question 1, compared to 31 in question 8. To assess the extent to which participants’ opinions were stable or subject to change upon further reflection, we examined the reasons behind their responses and categorized any shifts in opinion.

Among the 32 participants who initially supported continuous monitoring (Question 1), 24 maintained their positive stance, while 8 changed to a negative viewpoint. The reasons for these changes, collected as free-text responses, included concerns about redundancy (*n* = 3), potential dependency (*n* = 3), and conditional necessity (*n* = 2). Conversely, among the 34 participants who were initially against continuous monitoring, 7 shifted to a positive stance by the end of the survey, while the remaining 27 continued to express negative views. Their reasons for maintaining this negative stance included conditional necessity (*n* = 18), disinterest (*n* = 5), viewing the technology as a gimmick (*n* = 1), potential dependency (*n* = 1), reliance on an emergency button (*n* = 1), or choosing not to comment (*n* = 1).

Similarly, for questions 2 and 9, which explored participants’ concerns regarding cM, 61 participants initially reported no concerns about the concept. However, we observed shifts in concern levels throughout the survey. Specifically, 4 out of 5 participants who initially expressed concerns about continuous monitoring (Q2) overcame these concerns by the end of the survey (Q9). Conversely, 4 participants who started the survey without concerns developed apprehensions over time. These new concerns were related to measurement accuracy (*n* = 2), data security (*n* = 1), and potential diagnostic inaccuracies (*n* = 1).

Overall, 57 participants expressed no concerns or fears regarding continuous monitoring by the end of the survey, as indicated by their responses to Questions 2 and 9. Their reasons included disinterest (*n* = 4), a view of conditional necessity (*n* = 1), or seeing it as a gimmick (*n* = 1). Fifty-one participants did not provide specific comments on the matter.

These findings suggest that while the majority of participants maintained their initial stance, some revised their views after engaging with the survey. The bidirectional nature of these changes—both toward and away from a positive stance—indicates that participants’ opinions were not only influenced by their initial perceptions but also evolved based on reflection and additional information. This underscores the importance of understanding how stable or malleable initial opinions are and whether exposure to different perspectives can lead to reconsideration. Further discussion is warranted to explore the factors contributing to these opinion shifts and their implications for the acceptance of continuous monitoring technologies.

## Discussion

### Internet use

Digital health services offer individuals a wide range of features to assist them in actively participating, engaging, and managing health prevention and healthcare [21]. We aimed to assess digital participation among German geriatric hospital patients and their views on continuous vital sign monitoring using wearables.

According to the 2022 Annual Report on the Digital Society, 91% of the German population uses the internet. However, among individuals aged 65 and older, the proportion is lower at 66.5% [8]. Notably, this finding aligns with our survey results, where 68.2% of participating geriatric hospital patients affirmed their internet use.

Interestingly, 27 out of 42 (64%) of participants aged 80 and above in our study reported using the internet which is impressive. However, if we take into consideration also the nonparticipants this proportion was probably much lower and probably much closer to the 37.3% reported by Reissmann et al. in a study of very old adults in Germany, which included additionally individuals with cognitive impairments [17]. The higher percentage in our findings may be due to our study’s exclusion of patients with cognitive impairments. Additionally, our study was conducted in 2023, two years later than the Reissmann et al. study, meaning our participants aged 80 and older were, on average, born two years earlier.

When considering all potential participants in our study, not just survey participants, the proportion of internet users was approximately 30% (30/149), which is even lower than the 37.3% reported in the Reissmann study. This discrepancy can be attributed to the fact that the previous study only included survey participants.

These findings suggest that while internet usage among older adults is increasing, the actual number of users may be lower than indicated in previous reports.

If internet activity is considered dependent on gender, the proportion of male onliners (75%) is higher than their female counterparts (66%). This phenomenon is also evident in other studies [17, 18]. A plausible explanation could be rooted in traditional gender role expectations, where women may adopt a more reserved stance towards the internet due to societal norms [9].

Furthermore, the influence of education on internet usage is evident, with a positive correlation between higher levels of education and increased internet engagement noted in previous studies [8, 17]. This trend is reflected in the results derived from the present study with geriatric participants. Specifically, 53.1% of those with a lower level of education reported internet use, compared to 84.6% among those with a medium level of education. While there was a slight dip among the highly educated, with 75% being online users, this variance may be attributed to the limited number of participants in this subgroup.

According to an expert report on the Eighth Ageing Report of the Federal Government, the connection between education and internet use can be rooted in the broader social networks that highly educated individuals possess, thereby affording them enhanced digital technology support. Additionally, higher education is often linked to increased income, rendering this group of people more capable of affording the necessary technological equipment [9].

The underlying premise that internet usage is contingent upon variables like income, gender, and age is not exclusive to this study. Similar correlations have been substantiated in investigations conducted within the context of Germany [22] and Europe [23].

Among the participants who did not use the internet (31.8%, 21/66), more reasons were given for being offline than conditions for internet usage. The main reason cited was a lack of interest, shared by sixteen out of 21 participants, some of whom had relatives available to perform online tasks. Stimulating interest in self-use of the internet through informative and age-appropriate guidance could be beneficial in encouraging digital engagement among these individuals [20].

### Wearables use

To use wearables, having digital participation is essential, requiring suitable technical devices. While most geriatric participants used 3–4 devices, aligning with the national average [18], it’s crucial to note that certain devices like landline telephones are irrelevant for wearables. Wearables operation necessitates an internet-enabled device, leading many participants to limit usage to 1–2 devices. Eighteen out of 66 participants faced barriers due to lacking internet-enabled devices. A similar trend was observed in a report on the very elderly, where an average utilization of 1.5 devices per person aged over 80 was documented, with 20% of this demographic lacking ownership of any internet-enabled device [17]. Specific device usage revealed that 40/66 (60.6%) of geriatric participants used smartphones, and 6/66 (9.1%) adopted wearables, which is a similar proportion as reported in a 2022 study [17]. Beyond device ownership, actual internet utilization is crucial. Some offliners owned smartphones but chose not to go online due to a perceived lack of necessity, indicating a lack of willingness to use wearables.

#### User behaviour

The use of the internet for the participants varies greatly for different applications. In the present survey, for example, the vast majority (33/45; 73.3%) used it for communication by messaging services, specifically WhatsApp. Comparable surveys have also highlighted the prominence of communication via e-mail (82.2%) applications like WhatsApp (40.5%) [17] and as frequent internet applications. This kind of communication is a good requirement regarding digital consultations with the physician.

An additional aspect pertains to the entry of sensitive data into wearable apps, which is often required for health monitoring. For this purpose, participants were asked whether they would be willing to shop online and thus enter sensitive banking data on websites. The response revealed that less than a third agreed to this practice, underscoring a certain level of distrust among two-thirds of geriatric participants concerning data security. A certain distrust was also shown in the Digital Index, wherein 44% of participants expressed minimal trust in the companies behind the applications they used [18].

Since digitalization has received a boost from the coronavirus pandemic [18], we were interested in whether participants *perceived* their internet usage as being influenced by this development. In our study, 15.5% (7/45) of online users reported that their internet usage had been influenced moderately or strongly by the pandemic, based on their subjective assessment.

When asked how they experienced this change, 9 out of 45 (20%) online participants provided an answer: 5 described it as mainly positive, and 4 as balanced. None of the respondents evaluated the change as mainly negative.

A comparison with the findings of Reissmann et al. shows some similarities and differences. Their study found that 25.5% of the oldest-old reported some level of influence on their internet usage, though for most, this influence was rather minor to moderate. Among those who experienced a change, 17.8% perceived it as positive, while 15.3% described it as negative, citing reasons such as increased insecurity, pressure, and a lack of social contact [17]. In contrast, in our sample, none of the respondents evaluated the change as mainly negative.

#### Digital competence

The survey revealed that geriatric participants, both online and offline, demonstrated high proficiency with technical devices, with 95.6% (43/45) of online participants and 81% (17/21) of offline participants reporting comfort. This contrasts positively with a report on very old individuals, where 43% found using technical devices (very) challenging and only about a third found it (rather) not difficult [17]. This discrepancy between the studies can be attributed to the fact that we could not interview the nonparticipants on this matter, which may have skewed the results in a positive direction.

The majority of onliners expressed interest in technical devices (34/45; 75.6%) and considered them beneficial for everyday life (37/45; 82.2%), surpassing the levels reported for the very old (61.5% and 33% respectively) [17]. This openness to technological progress was further evident in online participants’ willingness for video consultations (27/45; 60%) and wearing wearables (22/45; 49%) for health data recording. Offliners, while less enthusiastic, still demonstrated a positive outlook toward technological progress, with (9/21; 42.9%) expecting everyday life simplification and 52.4% (11/21) anticipating personal benefits.

Beyond self-assessment, specific activities confirmed the digital skills of the onliner group. For instance, 38 out of 45 (84.4%) were able to take and send pictures with smartphones, and 29 out of 45 (64.4%) found tasks like completing online forms and managing passwords unproblematic. While their digital competence level, according to the Digital Index, was classified as rather low, participating onliners demonstrated solid skills in operating wearables, including creating accounts and inputting health data.

It is important to note that this group represents a positively selected sample of internet-active older adults in a clinical setting, which likely includes individuals who are more interested in and open to technology. Therefore, while these findings indicate that engaged older users can develop practical digital skills, they cannot be generalized to the entire older population. Instead, they highlight the potential for increased digital inclusion among those who are willing and able to engage with digital applications.

### Opinion regarding continuous monitoring

In this survey section, participants were asked if they could envision continuous monitoring and if they had any concerns. Notably, 53% (35/66) expressed a favourable disposition towards continuous monitoring. Concerns about data transfer to family doctors included redundancy and fear of premature diagnoses. Half (50%, 33/66) of the participants opposed video consultations due to the importance of personal contact with physicians, consistent with another study [24].

Encouragingly, 47% (31/66) were willing to share health data for research, but only 31.8% (21/66) would participate in a study due to time constraints and travel exhaustion. To overcome these barriers, providing targeted guidance and support within the clinical setting could help facilitate wearable usage and improve preparedness for home use. This suggests that a structured and supportive approach within healthcare facilities could enhance study participation.

### Limitations and strengths

A key finding, but also a major limitation of the study was that only 66 out of 149 potential participants agreed to be interviewed. These respondents represent a highly selective group of geriatric hospital patients. For patients who chose not to participate in the study, only their reasons for non-participation were collected. Fortunately, this allowed us to analyze the question of digital engagement to a level that may be representative for geriatric hospital patients without cognitive disabilities.

In the context of subgroup analyses, it is crucial to acknowledge the limitations posed by the small sample size and the high selectivity of the sample. This allows for the identification of trends but not definitive conclusions.

### Future research direction

The findings of this study highlight the need for wearable technologies that are tailored to the unique needs of geriatric patients. Given the increasing age and complexity of this patient population, future research should prioritize the development of systems that require minimal active patient involvement, thereby reducing the burden on individuals with limited digital competencies.

Additionally, it is crucial to address the fears and anxieties that patients may experience when using such devices. Future studies should explore strategies to mitigate these concerns, such as improved patient education, user-friendly interfaces, and clear communication about the benefits and limitations of wearable technologies.

Finally, based on the results of this study, specific guidelines for effective patient education and support should be developed. These guidelines could include recommendations for healthcare providers on how to communicate with geriatric patients about wearable technologies, as well as actionable steps for designing devices that are both effective and accessible.

## Conclusion

Despite its limitations, we were able to demonstrate that at least 30% (45/149) of potential participants in the geriatric hospital were proficient internet users with solid digital skills. Among the group of actual study participants, this proportion was significantly higher, reaching 68.2% (45/66). However, when considering the entire consecutively screened geriatric inpatient population, at least 22% (45/201) were confirmed as digitally competent. This proportion may be underestimated, as 52 patients were excluded based on study criteria, and their digital engagement remains unknown.

Notably, within the group of offline participants, 19% (4/21) indicated that they might adopt online usage if they perceived direct benefits. Whether these findings fully justify the optimistic conclusions drawn in the final statement is left to the reader’s interpretation. Nevertheless, they suggest potential avenues for exploring wearable-oriented studies in geriatric hospital settings. The integration of digital innovations could contribute to easing caregiver burdens while supporting high-quality patient care in the future.

## Electronic supplementary material

Below is the link to the electronic supplementary material.

Supplementary Material 1

## Data Availability

The datasets used and/or analysed during the current study are available from the corresponding author upon reasonable request.

## Abbreviations

SD: Standard deviation
SIS: Six-item-screen-test
SPS: Statistical package for the social sciences

## References

[1] Blüher S, Stein T, Schilling R, Grittner U, Kuhlmey A. Vermeidung von Pflegebedürftigkeit – Herausforderungen für Forschung und Praxis. Pflege-Report 2021. 2021 [cited 2024 Mar 14];91–102. Available from: https://link.springer.com/chapter/10.1007/978-3-662-63107-2_6

[2] Freund H. Altersmedizin und geriatrisches assessment. ÄP NeurologiePsychiatrie. 2013;1:24–6.

[3] Bonin H. Fachkräftemangel in der Gesamtperspektive. Pflege-Report 2019. 2020;61–9.

[4] Heger D, Wachstumsmarkt Pflege. Pflege-Report 2021. 2021 [cited 2024 Mar 14];145–56. Available from: https://link.springer.com/chapter/10.1007/978-3-662-63107-2_10

[5] Malwade S, Abdul SS, Uddin M, Nursetyo AA, Fernandez-Luque L, Zhu X, Katie K et al. Mobile and wearable technologies in healthcare for the ageing population. Comput Methods Programs Biomed. 2018 [cited 2024 Mar 14];161:233–7. Available from: https://pubmed.ncbi.nlm.nih.gov/29852964/10.1016/j.cmpb.2018.04.026 29852964

[6] DIP - Gesetz für eine bessere Versorgung durch Digitalisierung und Innovation. (Digitale-Versorgung-Gesetz - DVG). [cited 2024 Mar 14]. Available from: https://dip.bundestag.de/vorgang/gesetz-f%C3%BCr-eine-bessere-versorgung-durch-digitalisierung-und-innovation-digitale-versorgung-gesetz/251761?term=Digitale-Versorgung-Gesetz26;rows=2526;pos=1

[7] Meskó B, Görög M. A short guide for medical professionals in the era of artificial intelligence. NPJ Digit Med. 2020 [cited 2024 Mar 14];3(1). Available from: https://pubmed.ncbi.nlm.nih.gov/33043150/10.1038/s41746-020-00333-z PMC7518439 33043150

[8] D21-Digital-. Index 2020/2021 – Jährliches Lagebild zur Digitalen Gesellschaft.

[9] Ehlers A, Heß M, Frewer-Graumann S, Olbermann E, Stiemke P, Hagen C et al. Digitale Teilhabe und (digitale) Exklusion im Alter Expertisen zum Achten Altersbericht der Bundesregierung. 2020. https://www.achter-altersbericht.de/fileadmin/altersbericht/pdf/Expertisen/Expertise-FFG-Dortmund.pdf

[10] Wangler J, Jansky M. The role of gratifications in the process of adopting new media in higher age: Results of a qualitative study with very old users of digital media. Z Gerontol Geriatr. 2021 [cited 2024 Mar 14];54(8):781–8. Available from: https://link.springer.com/article/10.1007/s00391-020-01833-z 10.1007/s00391-020-01833-z PMC8636434 33369694

[11] Schmidt L. Technikhandhabung im höheren Alter. Technikhandhabung im höheren Alter. 2017.

[12] Berner, Frank. Endter Cordula, Hagen Christiane. Ältere Menschen und Digitalisierung. 2022.

[13] Fokkert MJ, Van Dijk PR, Edens MA, Abbes S, De Jong D, Slingerland RJ et al. Performance of the FreeStyle Libre Flash glucose monitoring system in patients with type 1 and 2 diabetes mellitus. BMJ Open Diabetes Res Care. 2017 [cited 2024 Mar 14];5(1):e000320. Available from: https://drc.bmj.com/content/5/1/e000320 10.1136/bmjdrc-2016-000320 PMC5316912 28243449

[14] Lubitz SA, Faranesh AZ, Selvaggi C, Atlas SJ, McManus DD, Singer DE et al. Detection of Atrial Fibrillation in a Large Population Using Wearable Devices: The Fitbit Heart Study. Circulation. 2022 [cited 2024 Mar 14];146(19):1415–24. Available from: https://pubmed.ncbi.nlm.nih.gov/36148649/10.1161/CIRCULATIONAHA.122.060291 PMC9640290 36148649

[15] Perez MV, Mahaffey KW, Hedlin H, Rumsfeld JS, Garcia A, Ferris T et al. Large-Scale Assessment of a Smartwatch to Identify Atrial Fibrillation. N Engl J Med. 2019 [cited 2024 Mar 14];381(20):1909–17. Available from: https://pubmed.ncbi.nlm.nih.gov/31722151/10.1056/NEJMoa1901183 PMC8112605 31722151

[16] Klebbe R, Steinert A, Müller-Werdan U. Wearables for older adults: Requirements, design, and user experience. Perspectives on Wearable Enhanced Learning (WELL): Current Trends, Research, and Practice. 2019;313–32.

[17] Reissmann M, Oswald V, Zank S, Tesch-Römer C. Digitale Teilhabe in der Hochaltrigkeit. 2022 [cited 2024 Mar 14];80. Available from: https://ceres.uni-koeln.de/forschung/d80

[18] D21-Digital-Index. 2022/2023 - Initiative D21. [cited 2024 Mar 14]. Available from: https://initiatived21.de/publikationen/d21-digital-index/2022-2023

[19] Krupp S, Schöne F, Balck F, Hofmann W, Willkomm M, Kasper J. Self-assessment of daily activities using the duruöz hand Index—Validation of the German translation. Z Gerontol Geriatr. 2022;55(2):99–104. 35190873 10.1007/s00391-022-02041-7

[20] Holetschek Birgit S, Anja K, Günther P, Hubert. Schmidmeir Lisa. netzwerk-altenhilfe.de. 2021. Befragung zur Digitalisierung bei Seniorinnen.

[21] Smahel D, Elavsky S, Machackova H. Functions of mHealth applications: A user’s perspective. Health Informatics J. 2019 [cited 2024 Mar 14];25(3):1065–75. Available from: https://journals.sagepub.com/doi/10.1177/1460458217740725 10.1177/1460458217740725 29121831

[22] Quittschalle J, Stein J, Luppa M, Pabst A, Löbner M, Koenig HH et al. Internet Use in Old Age: Results of a German Population-Representative Survey. J Med Internet Res. 2020 [cited 2024 Mar 14];22(11). Available from: https://pubmed.ncbi.nlm.nih.gov/33226351/10.2196/15543 PMC7685698 33226351

[23] König R, Seifert A. From online to offline and vice versa: change in internet use in later life across Europe. Front Sociol. 2020;5:489990. 10.3389/fsoc.2020.00004 PMC8022469 33869413

[24] Bevölkerungsumfrage. Future Health. 2018 https://www.pwc.de/de/gesundheitswesen-und-pharma/pwc-future-health-berichtsband.pdf

