# Supplementary material for: A monocentric prospective study investigating digital engagement among geriatric hospital patients
Source: BMC Geriatr. 2025 May 20;25:361. doi: 10.1186/s12877-025-05953-2 (PMC12090629; doi:10.1186/s12877-025-05953-2)
Supplement: Supplementary file 1 — Supplementary Material 1 [file 12877_2025_5953_MOESM1_ESM.pdf]

1. Demographic Data

1.1. Which gender do you feel you belong to?

- ○ male
- ○ female
- ○ diverse

1.2. In which year were you born? ______________________________

1.3. What is your highest level of education?

- ○ no school / low education
- ○ medium education
- ○ high education
- ○ Bachelor / Master / Diploma
- ○ other: ___________________________

1.4. Which profession have you had (for the longest time)?_______________________

2. Digital Participation

2.1. Digital devices and internet

2.1.1. Please say which of the following devices you use privately and how often.

	daily	several times a week	several times a month	never/ no comment
Landline phone				
Cell phone (without internet)				
Smartphone (with internet)				
Computer				
Laptop				
Tablet				
(Smart) TV				
Wearable (smart watch etc.)				

2.1.2. Do you use the internet?

- ○ No **[continue with question 2.1.3. for Offliners]**
  - ☐ I don't have access to the internet.
  - ☐ I'm not interested in the internet in general.
  - ☐ It's too complicated for me to deal with the internet.
  - ☐ Newspaper, book or radio is ok.
  - ☐ I have someone who does everything for me.
  - ☐ I don't want to ask for help.
  - ☐ I don't see any benefit/advantage in it.
  - ☐ I'm afraid for my personal information.
  - ☐ Other:____
  - ☐ No comment.

- ○ Yes, this type of internet **[skip this question for Offliners]**

  ☐ only internet (WIFI) at home.

○ daily	○ several times a week	○ several times a month	○ several times a year	○ never / no comment

  ☐ also mobile data or WIFI on the move.

○ daily	○ several times a week	○ several times a month	○ several times a year	○ never / no comment

2.1.3. Under which conditions would you use the internet in the future? **[Offliners]**

- ☐ If I had access to the internet.
- ☐ If I had a contact person, who shows me how it is working.
- ☐ If the use were easier.
- ☐ If I knew how to (better) protect my data.
- ☐ If I saw a clear benefit to me.
- ☐ Other: ____
- ☐ I don't know / no comment.

2.2. Digital Competence

2.2.1. How well do you get along with the devices indicated in 2.1.1. above?
→ turn on/off, operate: make calls, open apps…

O very good	O good	O bad	O very bad	O no comment

2.2.2. How well do you cope with the internet?
→ turn on/off the internet, search and filter information…

O very good	O good	O bad	O very bad	O no comment

2.3. Usage Profile (frequency and motivators)

2.3.1. Please indicate whether and how often you have used the following applications in the last 12 months.

	daily	several times a week	several times a month	never/ no comment
Communication with family and friends via messaging services (WhatsApp...)				
Communication with family and friends via video conferencing (Skype, Zoom...)				
Gaming apps, e.g. memory games...				
Netflix / streaming services | online libraries from TV programs like ZDF, ARD...				
Online shopping (groceries/clothes via direct bank transfer, PayPal...)				
Health care (health or fitness applications, such as counting steps or measuring blood glucose levels)				

2.3.2. Please indicate to what extent the following statements apply to you personally.

	applies fully	tends to apply	tends not to apply	applies not at all	no comment
I'm interested in technical things.					
I can take pictures and videos with my smartphone and send them to others.					
I can fill in forms online, e.g. Corona vaccination registration, pension forms...					
I can cope with the password assignment.					
Digital devices make my everyday life easier, e.g. blood pressure monitor for at home, talking watch, ...					
I have a contact person who can help me with questions about the internet.					

3. Opportunity of Digitalisation in geriatrics, using the example of continuous monitoring/surveillance

During your stay as an inpatient, your blood glucose level, blood pressure, heart rate/pulse, and/or heart rhythm will be monitored and documented around the clock (24h). These measurements are taken by small devices such as wristbands or sensor patches that you wear on your body. Through 24-hour monitoring, irregularities such as cardiac arrhythmias, lack of exercise, or diabetes can be detected and treated at an early stage.

3.1. Would you welcome 24h monitoring of your blood pressure, pulse, and/or blood glucose levels?

O yes	O no

Reason:

____

____

3.2. Do you have concerns about 24h monitoring of these values?

O yes	O no

Reason:

____

____

3.3. Would you be willing to take part in studies that perform 24h monitoring with digital devices?

O yes	O no

Reason:

____

____

3.4. Would you be willing to make your data available for research if it is pseudonymised?

O yes	O no

Reason:

____

____

3.5. Do you have confidence that your blood pressure, pulse, and/or blood glucose levels can be determined accurately with digital devices?

O yes	O no

Reason:

____

____

3.6. Would you be willing to connect digital devices to your smartphone?

→ You can then look at your values on your smartphone at any time.

O yes	O no

Reason:

____

____

3.7. Would you agree to transmit your data to your family doctor?

O yes	O no

Reason:

____

____

3.8. Do you think it would be beneficial if you had 24-hour monitoring of your blood pressure, pulse, and/or blood sugar levels?

O yes	O no

Reason:

____

____

3.9. Do you have any fears about having your blood pressure, pulse, and/or blood glucose levels monitored 24 hours a day?

O yes	O no

Reason:

____

____

4. Concluding Questions with a Mood Survey

4.1. To what extent do you agree with these statements on the use of digital devices?

	applies fully	tends to apply	tends not to apply	applies not at all	no comment
I can imagine being treated by a doctor via video consultation.					
I can imagine using a technical device to record my health data at home as well.					
I believe that I benefit overall from technological progress.					

4.2. How much did the Corona pandemic influence your internet use?

O not at all	O little	O medium	O strong	O very strong

4.3. How did you experience this change?

O mainly negative	O mainly balanced	O mainly positive
